# DNA methylation risk scores for depression, not today

**DOI:** 10.1192/j.eurpsy.2024.1218

**Published:** 2024-08-27

**Authors:** E. Van Assche, C. Hohoff, B. T. Baune

**Affiliations:** ^1^Klinik für Psychische Gesundheit, University of Münster, Münster, Germany; ^2^Department of Psychiatry; ^3^The Florey Institute of Neuroscience and Mental Health, University of Melbourne, Melbourne, Australia

## Abstract

**Introduction:**

After the success of polygenic risk scores (PRS) that embed a useful summary of genomic information in a comprehensive score, the wish to develop summary statistics for DNA methylation had become more pressing. Developing such a score faces challenges, as the score has to be specific and sensitive as well. Epidemiological research on DNA methylation and depression would benefit from such score.

**Objectives:**

Here, we test a score trained on incident depression (case-control), i.e., a list of published weights for particular CpGs, for its validity in the context of depression severity as measured using MADRS in our sample with depressed patients only.

**Methods:**

DNA methylation was assessed using the Illumina Infinium MethylationEPIC 850k BeadChip on a sample of 119 patients with a diagnosis of MDD. After data cleaning, 113 participants were included in the analysis (M_age_= 47 years, 57.98% women, M_MADRS_=27.7). Data processing was conducted using the RnBeads package. From the published reference for the overall sample, a list of 196 CpGs was provided, 170 of these were present in our dataset and used for the score. The list of non-smokers comprised 144 CpGs, of which 124 were available. The score per individual was built using M-values, using the formula: S(weight*DNA methylation value). The score was tested in association with depression and other typical confounders using multiple regression in R. Confounders included ancestry, BMI, age, sex, and 6 cell types. We tested both scores in our sample: smokers and non-smokers.

**Results:**

In contrast to our expectations, none of the regression analyses showed a significant association with depression (MADRS-score). Nonetheless, a significant association was seen with biological sex for both analysis (overall: p=0.036, non-smokers: p=0.026). A reduced model with only this predictor explained 5% and 4% of the variance of the summary score calculated (R^2^), respectively (overall: p=0.013; non-smokers: p=0.019). One of the ancestry components was marginally significant too in the non-smoker summary score (p=0.065). This was not the case anymore in the reduced model.

**Conclusions:**

Our results show that caution is still in place when using methylation risk scores as specificity and sensitivity might not yet be optimized. The score built for depression incidence does not seem fitting for depression severity at this moment. The use of DNA methylation, a marker that is generally sensitive to confounding factors, for a risk score, might pose more challenges in the context of reliable summary statistics, in particular also for cross-trait examination, which is currently a typical use of polygenic risk scores.

**Disclosure of Interest:**

None Declared

